# Current status of knowledge on the prevention of common infectious diseases among urban and rural populations: a cross-sectional study

**DOI:** 10.3389/fpubh.2026.1771435

**Published:** 2026-05-11

**Authors:** Jia-Ning Fan, Yuan-Kai Wang, Huan-Zheng Sun, Ping Hu, Yue Wang, Na Ban

**Affiliations:** 1 Department of Tuberculosis Clinic, Beijing Center for Disease Prevention and Control, Xinguokou Dongguang Hutong, Beijing, China; 2 Department of Rehabilitation Medicine, Beijing Fengtai You‘anmen Hospital, Beijing, China; 3 Department of Science and Education, Tianjin Institutes of Health Science, Huijin City, Tianjin, China; 4 Department of Research, China Pharmaceutical Innovation and Research Development Association, Beijing, China; 5 Department of Clinical Research, The First Hospital of Jilin University, Changchun, Jilin, China; 6 Zhejiang Tongpu Medical Technology Co., Ltd., Tongxiang/Jiaxing, Zhejiang, China

**Keywords:** common infectious diseases, community-based cross-sectional study, health literacy, preventive knowledge, urban–rural disparities, vaccination

## Abstract

**Background:**

Common infectious diseases continue to impose a considerable burden in community settings, and prevention depends largely on public knowledge, attitudes, and practices. Urban–rural and socioeconomic disparities may lead to unequal levels of preparedness. This study assessed the current status and determinants of knowledge regarding the prevention of common infectious diseases among community-dwelling adults in a municipal area.

**Methods:**

A cross-sectional survey was conducted using a multistage stratified cluster sampling approach. Community-dwelling adults aged ≥18 years from urban and rural districts were surveyed. A structured questionnaire was developed to assess sociodemographic factors, knowledge on disease transmission, prevention, and attitudes. Knowledge scores (0–17) were categorized as adequate (≥13) or inadequate (< 13). Descriptive statistics and logistic regression were used to analyze the data.

**Results:**

Of 872 residents approached, 859 valid questionnaires were analyzed. The mean knowledge score was 12.0 ± 3.1, with 31.9% of participants achieving adequate knowledge. Urban residents showed higher scores than rural residents. The most significant factors associated with adequate knowledge were urban residence [adjusted odds ratio (aOR) 1.65, *P* = 0.002], higher education, higher income, and younger age. Preventive practices, including vaccination, hand hygiene, and mask use, were suboptimal, with rural residents reporting lower adherence to preventive behaviors (*P* < 0.001).

**Conclusions:**

Community knowledge of common infectious disease prevention appeared to be moderate, with observable gaps in practical preventive behaviors and sociodemographic differences. These findings suggest that targeted, equity-focused health education strategies could help strengthen behavior-oriented prevention, particularly among older, rural, and lower-income populations.

## 1 Introduction

Infectious diseases remain a major contributor to global morbidity and mortality despite advances in diagnostics, therapeutics, and vaccination programs (1). In the post–coronavirus disease 2019 (COVID-19) era, the importance of sustained, community-level prevention has been reinforced not only for emerging pathogens, but also for respiratory, enteric, and vaccine-preventable infections that continue to impose a substantial public health burden (2). Population-based surveys consistently show that deficits in infectious disease–related knowledge, attitudes, and practices (KAP) cluster in socioeconomically disadvantaged groups and rural areas, and may contribute to persistent inequalities in infection risk and outcomes (3, 4). Understanding these patterns in the general adult population, rather than in disease-specific or clinical subgroups alone, is essential for designing targeted public health interventions. Health literacy, and more specifically infectious disease–specific health literacy (IDSHL), has emerged as a key determinant of individuals' ability to access, understand, and use information on infection prevention. Higher IDSHL is associated with better adherence to recommended preventive behaviors, more appropriate health-care–seeking, and improved self-management during outbreaks (5). Recent studies in rural and semi-urban settings have documented suboptimal IDSHL and infectious disease knowledge, with clear gradients by education, income, and household location, and have highlighted the weak correlation between knowledge and actual preventive practice (3, 6). Parallel work in children and adolescents has shown that infection-related knowledge and practice are strongly shaped by broader health literacy, electronic health literacy, and the social and educational environment, suggesting that social determinants operate across the life course (7, 8).

Vaccination remains a cornerstone of infectious disease prevention, yet adult immunization coverage is suboptimal in many settings. Surveys in clinical and community populations reveal substantial knowledge gaps and inconsistent uptake of recommended adult vaccines, even among high-risk groups and healthcare workers (9). National surveillance data indicate that coverage for most adult vaccines remains below target levels, with pronounced disparities by age, education, and socioeconomic status (10, 11). At the same time, the landscape of health information has become increasingly complex. Traditional mass media and primary care providers continue to disseminate infection-related messages, while digital platforms now constitute a major source of health information for many adults (12). Urban–rural differences in access to digital infrastructure, professional health services, and organized health education activities may further shape disparities in preventive knowledge (13, 14). Community-level infectious disease prevention relies heavily on public understanding of transmission mechanisms and protective behaviors (15). In routine public health practice in China, prevention messages are typically delivered through integrated education campaigns that address multiple nationally prioritized communicable diseases simultaneously rather than in isolation (16). These campaigns emphasize shared preventive strategies, such as vaccination, hand hygiene, respiratory etiquette, safe food and water practices, avoidance of high-risk exposure, and timely health-seeking behavior, that form the foundation of community-based infection control (17). Tuberculosis, HIV/AIDS, hepatitis B, and influenza are nationally notifiable diseases consistently included in public health promotion programs (18). Their selection in this study was based on their routine inclusion in community education initiatives rather than solely on incidence ranking (19). While differing in etiology and epidemiology, they collectively represent diverse transmission categories (respiratory, blood-borne, contact, and environmental pathways) commonly addressed in community education (20). This diversity was intended to reflect the breadth of preventive concepts communicated in routine practice and to assess general preventive literacy across transmission contexts, rather than to imply biological equivalence or to conduct disease-specific epidemiological comparisons.

Within this integrated public health context, there is a need for comprehensive, population-based assessments that examine overall preventive knowledge, domain-specific understanding of transmission routes and protective measures, vaccination attitudes and hygiene behaviors, and the use and perceived credibility of different information sources. Furthermore, the independent contributions of key sociodemographic factors, including age, sex, education, income, occupation, and place of residence, to adequate preventive knowledge remain incompletely characterized in general adult populations. The present cross-sectional study addresses these gaps by evaluating knowledge, preventive attitudes and practices, and information sources related to selected communicable diseases among urban and rural residents within a single municipal setting, and by identifying sociodemographic correlates of adequate knowledge to inform equity-oriented public health strategies.

## 2 Methods

### 2.1 Study design

This cross-sectional study was conducted to assess the current status of knowledge regarding the prevention of common infectious diseases among urban and rural residents in our municipality. The target population comprised community-dwelling adults residing in the sampled urban and rural communities. Inclusion criteria were: (1) being a registered or de facto permanent resident of the selected community; (2) age ≥18 years; (3) residence in the locality for at least 6 months prior to the survey; and (4) ability to communicate effectively and understand the questionnaire content. Individuals were excluded if they had (1) severe cognitive impairment or clinically diagnosed mental disorders that interfered with communication; (2) severe visual, hearing, or speech impairments preventing valid interview; (3) acute critical illness at the time of the survey; or (4) refusal or inability to provide informed consent. Before data collection, all participants received a clear explanation of the study objectives, procedures, potential risks and benefits, and confidentiality safeguards. Informed consent was obtained from all participants. The study was reviewed and approved by the hospital's ethics committee and conducted in accordance with relevant guidelines and the Declaration of Helsinki. All data were anonymized prior to analysis to ensure participant confidentiality.

### 2.2 Questionnaire development and content

The survey questionnaire was developed based on a review of domestic and international literature on infectious disease prevention and existing community-based KAP instruments. An expert panel comprising infectious disease clinicians, epidemiologists, and public health specialists drafted and refined the items to ensure appropriateness for urban and rural populations. The content focused on four nationally notifiable communicable diseases, tuberculosis, human immunodeficiency virus/acquired immunodeficiency syndrome (HIV/AIDS), hepatitis B, and influenza, which are routinely included in community health education programs. The questionnaire consisted of sections on sociodemographic characteristics, general knowledge of infection, routes of transmission, preventive measures, attitudes and behaviors, and sources of health information. Knowledge items addressed core transmission pathways (person-to-person, food- and water-borne, respiratory, vector-related) and foundational preventive strategies (vaccination, hygiene practices, respiratory etiquette, environmental sanitation, and personal protective measures). The knowledge section consisted of 17 items covering general concepts/clinical manifestations (6 items), transmission routes (5 items), and preventive measures (6 items). Items were designed to assess general preventive literacy across core transmission and prevention domains rather than disease-specific knowledge. Each correct response was awarded one point, yielding a total score ranging from 0 to 17. The scale demonstrated acceptable internal consistency (Cronbach's α = 0.82). Prior to the main survey, a pilot test was conducted in 40 community residents who met the eligibility criteria but were not included in the final analytic sample. Participants completed the questionnaire twice at a 2-week interval to assess test–retest reliability, yielding an intraclass correlation coefficient of 0.86. Minor wording refinements were made based on participant feedback to improve clarity and comprehension; no substantial changes to the questionnaire structure were required. Corrected item–total correlations ranged from 0.32 to 0.68, indicating adequate item discrimination. The detailed questionnaire structure and item content are provided in Supplementary Table S1.

### 2.3 Questionnaire administration and data collection

In this study, “common infectious diseases” was used as an operational public health term referring to selected communicable diseases routinely addressed in community-level health education programs in the study area. Tuberculosis, HIV/AIDS, hepatitis B, and influenza were included as representative nationally prioritized notifiable diseases with established preventive strategies. The focus was on assessing general knowledge of cross-cutting prevention principles rather than disease-specific characteristics. Before formal data collection, all investigators received standardized training on the study objectives, the intent and administration of each questionnaire item, interviewing techniques, and procedures for protecting participant privacy and data confidentiality. Training also emphasized the use of neutral language and standardized prompts to reduce interviewer bias. During field implementation, trained investigators conducted face-to-face interviews in participants' homes or at community health service facilities. Investigators provided clarification using predefined explanations, and recorded responses in a uniform manner to minimize misunderstanding, particularly among individuals with limited literacy. Questionnaire completion was monitored on site to identify and correct missing or inconsistent responses. Written informed consent was obtained from all participants prior to administration of the questionnaire.

### 2.4 Sampling and sample size

A multistage stratified cluster sampling strategy was adopted. First, all districts and counties in the study area were stratified by urban vs. rural status, and within each stratum, community health service centers (urban) in Fengtai District and township health clinics (rural) in Pinggu District were randomly selected as primary sampling units. In each selected community, households were chosen using systematic sampling based on household registration lists, and one eligible permanent resident aged ≥18 years was randomly selected from each household using simple random selection procedures. The required sample size was estimated using the single-proportion formula expressed in text as: *n* = Z^2^ × *p* × (1 – *p*) ÷ d^2^, where n is the required sample size, Z is the standard normal deviate corresponding to the desired confidence level, p is the expected proportion, and d is the allowable absolute error. A 95% confidence level was assumed (*Z* = 1.96), with an expected proportion of adequate knowledge of common infectious disease prevention of 0.50 (*p* = 0.50) in the absence of robust prior data, and an allowable error of 0.05 (*d* = 0.05). This yielded a minimum sample size of 384 participants. To account for the cluster sampling design, this number was multiplied by a design effect of 2.0 (*n* ≈ 768) and further increased by 10% to compensate for anticipated non-response or invalid questionnaires, resulting in a final target sample size of approximately 850 participants.

### 2.5 Statistical analysis

All statistical analyses were performed using IBM SPSS Statistics, version 28.0 (IBM Corp., Armonk, NY, USA). Continuous variables were summarized as mean ± standard deviation (SD) for approximately normally distributed data and as median with interquartile range (IQR) for skewed distributions. Between-group comparisons of continuous variables (urban vs. rural residents) were conducted using independent-samples *t* tests or Mann–Whitney *U* tests, as appropriate. Categorical variables were expressed as counts and percentages and compared using the chi-square (χ^2^) test; Fisher's exact test was applied when expected cell counts were small. The total knowledge score was treated as a continuous variable for descriptive purposes and dichotomized for regression analyses as “adequate” (≥13/17) vs. “inadequate” (< 13/17). The threshold corresponds to approximately 75% correct responses and was selected *a priori* as a commonly used benchmark in community-based KAP/health literacy research to indicate satisfactory knowledge. Sensitivity analyses using adjacent cut-offs yielded similar patterns of association. Univariate binary logistic regression was used to examine crude associations between adequate knowledge and candidate sociodemographic variables (age, sex, residence, education level, household income, and occupation), and crude odds ratios (ORs) with 95% confidence intervals (CIs) were estimated. Variables with *P* < 0.10 in univariate analyses and those considered clinically or conceptually relevant were entered into a multivariable logistic regression model to identify independent predictors, and adjusted ORs (aORs) with 95% CIs were reported. Model discrimination was assessed using the area under the receiver operating characteristic curve (AUC), with 95% CI estimated by the nonparametric DeLong method. Calibration was evaluated using the Hosmer–Lemeshow goodness-of-fit test and calibration plots. Internal validation was performed using 10-fold cross-validation, and the mean AUC with standard deviation across folds was calculated to assess model stability. All tests were two-sided, and *P* < 0.05 was considered statistically significant.

## 3 Results

### 3.1 Participant recruitment and response rate

A total of 872 community-dwelling residents identified through the multistage stratified cluster sampling procedure were approached in the selected urban and rural communities during household visits and routine contacts at community health service facilities. Of these, 6 individuals declined participation, mainly due to lack of interest or time constraints, and 866 residents provided written informed consent and completed the questionnaire. All returned questionnaires were screened for data quality according to predefined criteria. Seven questionnaires were excluded from the analysis because of substantial missing data (more than 20% of key sociodemographic or knowledge items left unanswered, *n* = 5) or obvious logical inconsistencies in responses (e.g., reporting never having heard of infectious diseases while simultaneously providing detailed answers to specific prevention items, *n* = 2). Consequently, 859 questionnaires were considered valid and included in the final analysis, corresponding to a crude completion rate of 99.3% (866/872) and an overall valid response rate of 98.5% (859/872).

### 3.2 Sociodemographic characteristics of participants

Among the 859 participants included in the final analysis, 430 (50.1%) were urban residents and 429 (49.9%) were rural residents. The overall mean age was 47.2 ± 14.2 years, with urban residents being slightly younger than rural residents (45.8 ± 13.3 vs. 48.6 ± 15.0 years; *t* = −2.82, *P* = 0.005). The median duration of residence in the current community was 13.1 years (IQR 8.3–18.9), and was longer in rural than in urban areas [14.6 (IQR 9.9–20.6) vs. 11.7 (IQR 7.2–17.3) years; Mann–Whitney *U* = 71,964, *P* < 0.001]. The sex distribution was balanced (49.9% male and 50.1% female) and did not differ significantly between urban and rural residents (*P* = 0.759). In contrast, education level and occupation differed significantly by place of residence (both *P* < 0.001), with higher educational attainment and a greater proportion of office/professional staff in urban areas, and a predominance of farmers in rural areas. Monthly household income was also higher among urban residents, who more frequently reported income above the local average, whereas rural residents more often reported income below the local average (*P* < 0.001). Marital status and health insurance coverage were similar between urban and rural participants (both *P* > 0.70) (Table 1).

**Table 1:** Sociodemographic characteristics of participants by place of residence.

Characteristic	Total (*n* = 859)	Urban (*n* = 430)	Rural (*n* = 429)	Test statistic
Age, years				
Mean ± SD	47.2 ± 14.2	45.8 ± 13.3	48.6 ± 15.0	*t* = −2.82
Sex				
Male	429 (49.9%)	212 (49.3%)	217 (50.6%)	χ^2^ = 0.09
Female	430 (50.1%)	218 (50.7%)	212 (49.4%)	
Education level				
Primary school or below	158 (18.4%)	46 (10.7%)	112 (26.1%)	χ^2^ = 64.93
Junior high school	267 (31.1%)	115 (26.7%)	152 (35.4%)	
Senior high/vocational school	234 (27.2%)	131 (30.5%)	103 (24.0%)	
College or above	200 (23.3%)	138 (32.1%)	62 (14.5%)	
Occupation				
Farmer	174 (20.3%)	7 (1.6%)	167 (38.9%)	χ^2^ = 222.93
Worker/manual labor	166 (19.3%)	75 (17.4%)	91 (21.2%)	
Office/professional staff	200 (23.3%)	151 (35.1%)	49 (11.4%)	
Self-employed/small business	108 (12.6%)	68 (15.8%)	40 (9.3%)	
Student	59 (6.9%)	43 (10.0%)	16 (3.7%)	
Unemployed/retired/other	152 (17.7%)	86 (20.0%)	66 (15.4%)	
Marital status				
Single	188 (21.9%)	94 (21.9%)	94 (21.9%)	χ^2^ = 0.11
Married/cohabiting	586 (68.2%)	292 (67.9%)	294 (68.5%)	
Divorced/widowed	85 (9.9%)	44 (10.2%)	41 (9.6%)	
Monthly household income				
Below local average	301 (35.0%)	93 (21.6%)	208 (48.5%)	χ^2^ = 89.64
Around local average	332 (38.6%)	182 (42.3%)	150 (35.0%)	
Above local average	158 (18.4%)	120 (27.9%)	38 (8.9%)	
Not sure/refuse to answer	68 (7.9%)	35 (8.1%)	33 (7.7%)	
Health insurance status				
Insured	814 (94.8%)	409 (95.1%)	405 (94.4%)	χ^2^ = 0.10
Uninsured	45 (5.2%)	21 (4.9%)	24 (5.6%)	
Duration of residence, years				
Median (IQR)	13.1 (8.3–18.9)	11.7 (7.2–17.3)	14.6 (9.9–20.6)	*U* = 71964 c

SD, standard deviation; IQR, interquartile range; χ^2^, chi-square; *U*, Mann–Whitney *U* statistic.

### 3.3 Overall knowledge of common infectious disease prevention

The total knowledge score for common infectious disease prevention was calculated as the sum of correct answers to 17 knowledge items (6 general knowledge/clinical manifestations, 5 routes of transmission, and 6 preventive measures), with one point awarded for each correct response (score range 0–17). Among the 859 participants, the mean total knowledge score was 12.0 ± 3.1, with a median of 12 [interquartile range (IQR) 10–14] and a range of 3–17. Based on the total score, participants were categorized into three knowledge levels: low (0–8 points), moderate (9–12 points), and high (13–17 points). Overall, 207 (24.1%) participants were classified as having low knowledge, 378 (44.0%) as having moderate knowledge, and 274 (31.9%) as having high knowledge. Using a threshold of ≥13 points to define adequate knowledge, 274 participants (31.9%) met the criterion for adequate knowledge, whereas 585 (68.1%) had inadequate knowledge (Table 2).

**Table 2:** Overall distribution of total knowledge scores and knowledge levels (*n* = 859).

Measure	Value
Possible score range (number of items)	0–17 (17 items)
Observed score range	3–17
Mean ± SD of total knowledge score	12.0 ± 3.1
Median (IQR) of total knowledge score	12 (10–14)
Knowledge level categories a	*n* (%)
Low (0–8 points)	207 (24.1%)
Moderate (9–12 points)	378 (44.0%)
High (13–17 points)	274 (31.9%)
Dichotomized knowledge level	*n* (%)
Inadequate knowledge (< 13 points)	585 (68.1%)
Adequate knowledge (≥13 points)	274 (31.9%)

SD, standard deviation; IQR, interquartile range.

### 3.4 Knowledge in specific domains

Domain-specific analyses were performed for three knowledge domains: (1) general knowledge and clinical manifestations (6 items), (2) routes of transmission (5 items), and (3) preventive measures (6 items). The mean domain scores were 4.6 ± 1.3 (possible range 0–6) for general knowledge and clinical manifestations, 3.7 ± 1.2 (range 0–5) for routes of transmission, and 3.6 ± 1.5 (range 0–6) for preventive measures. These findings suggest relatively better understanding of basic concepts and transmission pathways and comparatively weaker knowledge regarding specific preventive practices. Item-level correct response rates further highlighted specific gaps. In the general knowledge domain, most participants correctly recognized that infectious diseases are caused by pathogenic microorganisms (B1, 88.6%), can spread from person to person (B2, 84.5%), and that vaccination is an important preventive measure (B6, 81.4%). However, only 57.9% correctly rejected the statement that “once infected, all infectious diseases are impossible to prevent from spreading to others” (B4), indicating incomplete understanding of the impact of early isolation and treatment.

In the routes of transmission domain, correct response rates were high for respiratory droplet transmission (C3, 81.7%) and close contact transmission (C1, 79.5%), but lower for vector-borne transmission (C4, 64.3%) and blood-borne transmission via shared needles (C5, 69.8%), suggesting that less visible transmission pathways are not fully appreciated. In the preventive measures' domain, knowledge appeared weakest. Although 70.0% correctly identified vaccination according to national immunization schedules as an important preventive strategy (D4), only 65.0% recognized the role of hand hygiene (D1) and 61.0% the benefit of mask use in crowded or poorly ventilated places (D2). Awareness of the importance of environmental sanitation (D5, 54.9%) and avoiding close contact with symptomatic individuals (D6, 48.0%) was particularly limited. These results indicate that health education should prioritize concrete preventive behaviors, especially environmental and interpersonal measures, to strengthen community-level infection prevention (Table 3).

**Table 3:** Domain-specific knowledge scores and correct response rates for individual items.

Domain	Item code	Item (abbreviated description)	Correct responses *n* (%)
General knowledge and clinical manifestations (6 items; possible score 0–6)			
Domain score, mean ± SD	–	–	4.6 ± 1.3
	B1	Infectious diseases caused by microorganisms	761 (88.6%)
	B2	Common infectious diseases can spread person to person	726 (84.5%)
	B3	Some infectious diseases have no obvious early symptoms	593 (69.0%)
	B4	“Once infected, all infectious diseases are impossible to prevent from spreading to others” (false)	497 (57.9%)
	B5	Early diagnosis and treatment reduce complications	654 (76.1%)
	B6	Vaccination is an important preventive measure	699 (81.4%)
Routes of transmission (5 items; possible score 0–5)			
Domain score, mean ± SD	–	–	3.7 ± 1.2
	C1	Transmission through close contact with infected persons	683 (79.5%)
	C2	Transmission via contaminated food or drinking water	626 (72.9%)
	C3	Respiratory droplets spread infection when coughing or sneezing	702 (81.7%)
	C4	Transmission through vectors such as mosquitoes or ticks	552 (64.3%)
	C5	Risk of infection from sharing needles/syringes	600 (69.8%)
Preventive measures (6 items; possible score 0–6)			
Domain score, mean ± SD	–	–	3.6 ± 1.5
	D1	Handwashing with soap helps prevent infection	558 (65.0%)
	D2	Mask use in crowded or poorly ventilated places reduces respiratory infection	524 (61.0%)
	D3	Properly cooked food and safe water reduce intestinal infections	550 (64.0%)
	D4	Vaccination according to national schedules prevents certain infections	601 (70.0%)
	D5	Environmental sanitation helps reduce spread of infectious diseases	472 (54.9%)
	D6	Avoiding close contact with symptomatic individuals helps prevent infection	412 (48.0%)

SD, standard deviation.

### 3.5 Factors associated with adequate knowledge

Adequate knowledge of common infectious disease prevention was defined as a total knowledge score ≥13 points; 274 participants (31.9%) met this criterion, while 585 (68.1%) had inadequate knowledge. In univariate logistic regression analyses, urban residence, higher education, higher monthly household income, and several non-farming occupations were positively associated with adequate knowledge. Compared with rural residents, urban residents had higher odds of adequate knowledge [crude odds ratio (OR) 2.04, 95% confidence interval (12) 1.52–2.74; *P* < 0.001]. Using primary school or below as the reference, the crude OR increased across education categories and reached 7.04 (95% CI 4.08–12.14; *P* < 0.001) in participants with college or higher education. Participants with income above the local average also had higher odds of adequate knowledge than those with income below the local average (crude OR 4.12, 95% CI 2.70–6.28; *P* < 0.001). Office/professional staff, self-employed individuals, and students showed higher crude odds than farmers, whereas sex and age exhibited only weak or non-significant associations in univariate analyses (Table 4).

**Table 4:** Univariate and multivariable logistic regression analyses of factors associated with adequate knowledge of common infectious disease prevention.

Predictor	Category	Adequate *n* (%)	Inadequate *n* (%)	Crude OR (95% CI)	P value	Adjusted OR a (95% CI)	*P* value
Age	Per 10-year increase	–	–	0.92 (0.84–1.00)	0.056	0.88 (0.79–0.98)	0.019
Sex	Male	128 (29.8)	301 (70.2)	1.00 (reference)	–	1.00 (reference)	–
	Female	146 (34.0)	284 (66.0)	1.21 (0.91–1.61)	0.196	1.05 (0.77–1.42)	0.756
Place of residence	Rural	104 (24.2)	325 (75.8)	1.00 (reference)	–	1.00 (reference)	–
	Urban	170 (39.5)	260 (60.5)	2.04 (1.52–2.74)	< 0.001	1.65 (1.20–2.28)	0.002
Education level	Primary school or below	20 (12.7)	138 (87.3)	1.00 (reference)	–	1.00 (reference)	–
	Junior high school	65 (24.3)	202 (75.7)	2.22 (1.29–3.83)	0.004	1.50 (0.97–2.30)	0.068
	Senior high/vocational school	88 (37.6)	146 (62.4)	4.16 (2.43–7.13)	< 0.001	2.05 (1.32–3.17)	0.001
	College or above	101 (50.5)	99 (49.5)	7.04 (4.08–12.14)	< 0.001	2.85 (1.77–4.58)	< 0.001
Monthly household income	Below local average	60 (19.9)	241 (80.1)	1.00 (reference)	–	1.00 (reference)	–
	Around local average	110 (33.1)	222 (66.9)	1.99 (1.38–2.86)	< 0.001	1.32 (0.95–1.82)	0.095
	Above local average	80 (50.6)	78 (49.4)	4.12 (2.70–6.28)	< 0.001	1.90 (1.29–2.79)	0.001
	Not sure/refuse to answer	24 (35.3)	44 (64.7)	2.19 (1.24–3.88)	0.007	1.38 (0.87–2.18)	0.174
Occupation	Farmer	35 (20.1)	139 (79.9)	1.00 (reference)	–	1.00 (reference)	–
	Worker/manual labor	45 (27.1)	121 (72.9)	1.48 (0.89–2.45)	0.130	1.12 (0.75–1.67)	0.579
	Office/professional staff	90 (45.0)	110 (55.0)	3.25 (2.04–5.17)	< 0.001	1.39 (0.92–2.10)	0.118
	Self-employed/small business	40 (37.0)	68 (63.0)	2.34 (1.36–4.00)	0.002	1.41 (0.86–2.31)	0.171
	Student	30 (50.8)	29 (49.2)	4.11 (2.19–7.72)	< 0.001	1.78 (0.95–3.33)	0.074
	Unemployed/retired/other	34 (22.4)	118 (77.6)	1.14 (0.67–1.95)	0.619	1.02 (0.62–1.67)	0.939

OR, odds ratio; aOR, adjusted odds ratio; CI, confidence interval.

In the multivariable model including age, sex, place of residence, education level, monthly household income, and occupation, urban residence remained independently associated with adequate knowledge [adjusted OR (aOR) 1.65, 95% CI 1.20–2.28; *P* = 0.002]. A clear graded association was observed for education: compared with primary school or below, senior high/vocational education (aOR 2.05, 95% CI 1.32–3.17; *P* = 0.001) and college or above (aOR 2.85, 95% CI 1.77–4.58; *P* < 0.001) were both significant predictors of adequate knowledge. Participants with income above the local average were also more likely to have adequate knowledge than those with income below the local average (aOR 1.90, 95% CI 1.29–2.79; *P* = 0.001). Increasing age was associated with slightly lower odds of adequate knowledge (aOR per 10-year increase 0.88, 95% CI 0.79–0.98; *P* = 0.019). Sex and occupation categories were not independently associated with adequate knowledge after adjustment (Table 4). The final model demonstrated acceptable discrimination (area under the ROC curve 0.73, 95% CI 0.69–0.77) and adequate calibration based on the Hosmer–Lemeshow goodness-of-fit test (*P* = 0.47).

### 3.6 Internal validation and calibration

**Figure 1:**
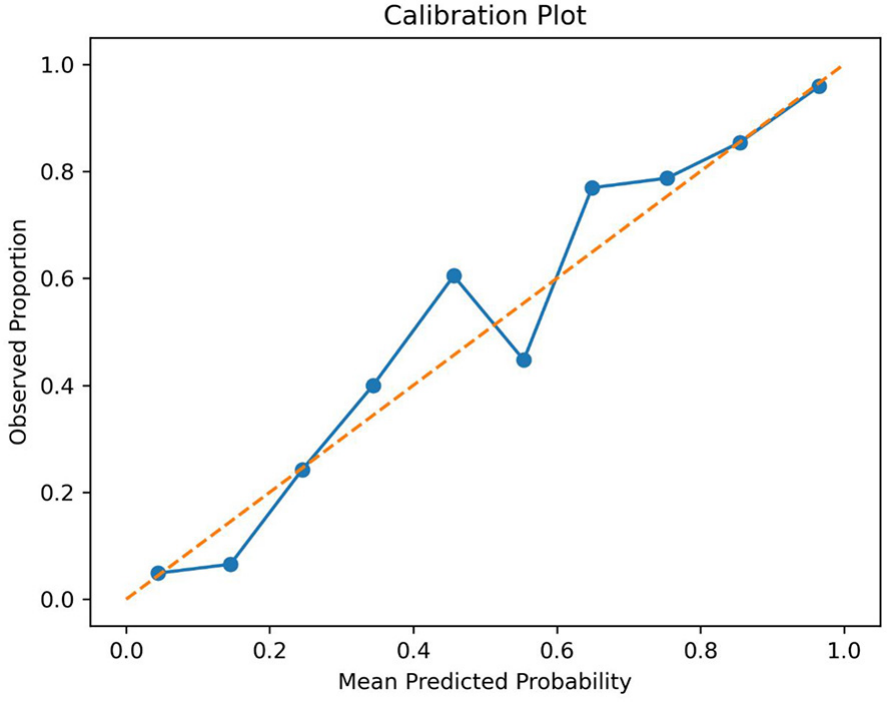
Calibration plot for the prediction model of adequate knowledge.

Internal validation using 10-fold cross-validation yielded AUC values ranging from 0.71 to 0.75, with a mean ± SD of 0.73 ± 0.01 (Supplementary Table S2), indicating stable moderate discrimination. The calibration plot demonstrated reasonable agreement between predicted and observed probabilities (Figure 1).

### 3.7 Preventive attitudes and practices

Regarding vaccination, 670 of 859 participants (78.0%) agreed or strongly agreed that adult vaccination is an important measure for preventing common infectious diseases, with a higher proportion among urban residents than rural residents (83.7% vs. 72.3%). Similarly, 636 participants (74.0%) agreed or strongly agreed that handwashing with soap is essential for preventing infection, and 601 (70.0%) endorsed the importance of mask use in crowded or poorly ventilated places, again with slightly higher proportions among urban residents. In terms of actual behaviors, 447 participants (52.0%) reported having received at least one recommended adult vaccine (e.g., seasonal influenza or other adult vaccines) in the past 3 years, with a substantial urban–rural difference (60.0% vs. 44.1%, *P* < 0.001). Regular hand hygiene was more common than vaccination: 584 participants (68.0%) reported that they “often” or “always” wash their hands with soap before eating or after using the toilet, with higher prevalence in urban than in rural residents (73.0% vs. 63.0%, *P* = 0.003). Mask use during epidemic seasons remained suboptimal, with 507 participants (59.0%) indicating that they “often” or “always” wear a mask in crowded indoor places, and again more frequent practice in urban areas (64.9% vs. 53.1%, *P* = 0.002). By contrast, willingness to seek timely medical care when experiencing persistent fever, cough, or diarrhea was high in both groups: 696 participants (81.0%) reported being “likely” or “very likely” to seek care at a medical institution, with no statistically significant difference between urban and rural residents (84.7% vs. 77.4%, *P* = 0.061) (Table 5).

**Table 5:** Attitudes and preventive practices related to infectious disease prevention by place of residence.

Indicator	Total (*n* = 859)	Urban (*n* = 430)	Rural (*n* = 429)	*P* value
Attitudes (likert-scale items)				
Agree/strongly agree that adult vaccination is important	670 (78.0%)	360 (83.7%)	310 (72.3%)	< 0.001
Agree/strongly agree that handwashing with soap is essential	636 (74.0%)	325 (75.6%)	311 (72.5%)	0.289
Agree/strongly agree that mask use in crowded/poorly ventilated places is important	601 (70.0%)	305 (70.9%)	296 (69.0%)	0.546
Likely/very likely to seek medical care for persistent fever/respiratory or diarrheal symptoms	696 (81.0%)	364 (84.7%)	332 (77.4%)	0.061
Preventive practices (behavioral indicators)				
Ever received at least one recommended adult vaccine in past 3 years b	447 (52.0%)	258 (60.0%)	189 (44.1%)	< 0.001
Often/always wash hands with soap before eating or after using the toilet	584 (68.0%)	314 (73.0%)	270 (63.0%)	0.003
Often/always wear a mask in crowded indoor places during epidemic seasons	507 (59.0%)	279 (64.9%)	228 (53.1%)	0.002

### 3.8 Sources of information on infectious disease prevention

With regard to sources of information, most participants reported obtaining infectious disease prevention messages from multiple channels, with notable differences between urban and rural residents. Overall, 617 of 859 participants (71.8%) indicated that they had received information from television or radio in the past year, 559 (65.1%) from internet health websites, 525 (61.1%) from social media platforms, 472 (55.0%) from healthcare professionals, 352 (41.0%) from community health education activities, and 333 (38.8%) from family members or friends. Urban residents were more likely than rural residents to use internet websites (72.3% vs. 57.8%) and social media (68.4% vs. 53.9%) as information sources, whereas rural residents more frequently cited community health education activities and family or friends. When asked to identify their single most trusted source of information, 266 participants (31.0%) selected healthcare professionals, followed by television or radio (24.7%), social media (18.2%), internet health websites (16.1%), and community health education activities (7.0%).

## 4 Discussion

This cross-sectional study provides a comprehensive assessment of knowledge, attitudes, and preventive practices related to common infectious diseases among urban and rural residents in a large municipal setting. Overall knowledge was moderate (mean score 12.0/17), yet only 31.9% of participants met the predefined threshold for adequate knowledge. Domain-level analysis revealed a clear gradient, with relatively higher performance in general concepts and transmission routes than in preventive measures (21). Although most participants recognized basic etiological principles and common transmission pathways, correct response rates were lower for concrete behavioral measures such as environmental sanitation and avoidance of close contact with symptomatic individuals. Urban residents consistently demonstrated higher knowledge levels and more frequent engagement in preventive behaviors than rural residents. Multivariable analysis further identified urban residence, higher education, higher household income, and younger age as independent predictors of adequate knowledge. The predictive model showed moderate discrimination and stable internal validity, suggesting that these sociodemographic factors explain a meaningful, though incomplete, proportion of variance in preventive literacy.

The observed pattern highlights a structural gap between conceptual awareness and operational preventive competence. While abstract knowledge regarding transmission mechanisms appears relatively well internalized, actionable behaviors, particularly those requiring sustained environmental or interpersonal adjustments, remain less consistently understood. This discrepancy is epidemiologically relevant, as community-level transmission is often driven by routine social interactions and environmental exposures rather than rare or highly specialized risks. The pronounced urban–rural differences in both knowledge and reported behaviors likely reflect disparities in educational attainment, income distribution, and access to diversified health information channels, as urban residents more frequently reported reliance on digital and professional sources. The inverse association between age and adequate knowledge further suggests potential generational differences in information acquisition and health literacy, particularly in rapidly evolving digital communication environments. Although the model demonstrated acceptable calibration and moderate discrimination (AUC 0.73), the remaining unexplained variance indicates that additional contextual factors, such as trust in public health messaging, prior exposure to community campaigns, or digital literacy, may play important roles. Together, these findings support the need for stratified, behavior-oriented health education strategies that prioritize practical implementation guidance and address structural inequities in information access (22, 23).

The multivariable model identified residence, education, income, and age as independent correlates of adequate knowledge, highlighting structural determinants of preventive literacy. Urban residence remained significant after adjustment (aOR 1.65), and education showed a graded association (senior high/vocational aOR 2.05; college or above aOR 2.85 vs. primary or below), suggesting that both educational attainment and the informational environment contribute to knowledge disparities. Higher household income also predicted adequate knowledge (aOR 1.90), plausibly reflecting greater capacity to access health services, digital resources, and preventive commodities (24). Increasing age was inversely associated with adequate knowledge (aOR 0.88 per 10 years), indicating that older adults may require tailored communication strategies that address health literacy, cognitive load, and preferred media. Although model discrimination was moderate (AUC 0.73; stable across 10-fold cross-validation, mean 0.73 ± 0.01) and calibration was acceptable (Hosmer–Lemeshow *P* = 0.47; calibration plot showed reasonable agreement), these performance metrics also imply that unmeasured factors, such as prior exposure to community education programs, trust in health authorities, digital literacy, and prior vaccination experiences, likely explain additional variance. Taken together, the findings support a stratified public health approach: strengthening rural outreach and lower-education groups with simplified, behaviorally specific content, leveraging trusted healthcare professionals as primary messengers, and aligning communication channels with urban–rural preferences to improve uptake of preventive practices (6, 25, 26).

The present findings are consistent with a growing body of evidence indicating that infectious disease–related knowledge remains unevenly distributed across populations and is strongly shaped by socioeconomic and contextual factors. Community-based surveys in diverse settings have similarly documented that, although general awareness may reach moderate levels, actionable preventive competence is often limited and concentrated among individuals with higher education and income. For example, Zhao et al. (21) reported that infectious disease–specific health literacy in China improved in the post–COVID-19 period but continued to exhibit clear gradients by urban–rural residence and socioeconomic status. Similarly, Banda Aron et al. (27) observed substantial deficiencies in knowledge of snakebite prevention and first aid in rural Malawi, even among community health workers, underscoring the persistent gap between exposure to information and operational understanding. In non-endemic settings, Ramos-Rincón et al. (28) further demonstrated that practicing physicians may exhibit incomplete knowledge of disease transmission and prevention, reinforcing that awareness does not necessarily translate into comprehensive preventive competence. Our results align with this broader structural pattern: while conceptual knowledge of transmission mechanisms was relatively well internalized, specific preventive behaviors, particularly those requiring sustained environmental or interpersonal adjustments, were less consistently understood. The independent associations observed for education, income, residence, and age suggest that preventive knowledge is embedded within broader informational and social infrastructures. As noted by Zhao et al., improvements in knowledge following intensive public health campaigns may not eliminate persistent sociodemographic disparities. The urban–rural differences identified in our study likely reflect not only socioeconomic inequalities but also differential access to diversified information channels and digital communication environments. Moreover, consistent with post-pandemic observations, behavior-oriented practices such as hand hygiene and mask use appear particularly vulnerable to erosion once perceived risk declines. By simultaneously examining multiple common infectious diseases within a unified analytic framework, the present study extends existing literature by demonstrating that structural determinants and behavioral knowledge gaps coexist at the community level, thereby highlighting the need for sustained, equity-oriented, and behavior-specific health education strategies rather than episodic, crisis-driven messaging.

The observed gaps in knowledge, especially regarding concrete preventive behaviors and less visible transmission routes, have direct implications for the design of community-based health education. Interventions should prioritize residents with lower education and income, older adults, and rural populations, using tailored materials that emphasize practical, actionable messages on hygiene, environmental sanitation, safe contact practices, and timely care-seeking (21). Strengthening collaboration between primary healthcare providers, community health workers, and local media channels may help to ensure that accurate, comprehensible information reaches sociodemographically disadvantaged groups and is reinforced over time rather than only during outbreak periods (24). The composite knowledge score was constructed as an operational index for this study to summarize participants' responses across multiple prevention-related items. It was designed to capture general cross-cutting preventive knowledge rather than disease-specific expertise. Although knowledge of different transmission routes represents distinct conceptual domains, the composite score was used as a pragmatic summary measure for analytic purposes (6, 25, 26). To provide additional granularity, we also reported domain-specific results for general knowledge, transmission routes, and preventive measures. We acknowledge that the unusually high response rates of 99.3% (crude completion rate) and 98.5% (valid response rate) observed in this study benefited from the assistance of volunteers and social workers at the selected institutions. Volunteers and social workers played a key role in facilitating the survey process, ensuring effective participant engagement, and minimizing non-responses. However, we recognize the potential for selection bias, interviewer influence, or the exclusion of non-contact households (29). While these factors were managed to the best of our ability, we will provide a more detailed explanation of our handling of refusals, replacement sampling, and non-response analysis in future studies to address these concerns. Household-level refusal rates were not systematically documented during fieldwork. Although the overall individual-level refusal proportion was low, the lack of detailed household-level records precludes formal assessment of non-response bias. If non-participating households differed systematically from participating households in sociodemographic characteristics or health awareness, external validity may be affected (30). However, given the very small number of refusals, the potential influence on generalizability is likely modest, although it cannot be entirely excluded.

Several limitations warrant consideration. First, the cross-sectional design precludes causal inference, and the reported associations represent correlations rather than temporal relationships. Second, although a multistage stratified cluster sampling strategy was employed, regression analyses were conducted at the individual level without explicit adjustment for clustering, and intraclass correlation coefficients were not estimated. As a result, observations within clusters may not have been fully independent, and variance estimates may have been affected by intra-cluster correlation, potentially leading to underestimated standard errors and slightly narrower confidence intervals. Sampling weights were not applied because equal selection probability within sampled clusters was assumed, which may limit population-level generalizability. The prespecified design effect was used solely for sample size estimation and was not incorporated into regression modeling. Third, the high response rate, while facilitating completeness, may introduce participation bias if non-responding households systematically differed from respondents; formal non-response analysis was not conducted. In addition, knowledge was operationalized as a composite index of cross-cutting preventive items rather than disease-specific constructs, which may obscure condition-specific variation. Preventive behaviors were self-reported and may be subject to recall and social desirability bias. Finally, residual unexplained variance suggests that unmeasured contextual factors, including digital literacy, trust in public health institutions, and prior exposure to health campaigns, may contribute to knowledge disparities. Future research should employ longitudinal designs to clarify temporal dynamics, incorporate multilevel modeling and sampling weights to improve population-level inference, and conduct disease-specific and mixed-methods investigations to better understand structural and behavioral barriers to sustained preventive practices. Intervention studies evaluating behavior-focused educational strategies and objective health outcomes are warranted to strengthen the evidence base for equity-oriented public health policy.

## 5 Conclusion

In this municipal community-based sample, knowledge of common infectious disease prevention was moderate, with fewer than one-third of residents meeting the prespecified criterion for adequate knowledge and notable gaps in several practice-oriented prevention items. Urban residence, higher educational attainment, higher household income, and younger age were associated with higher knowledge levels. These results might indicate the need for targeted, equity-oriented health education in similar community settings, with an emphasis on strengthening practical, behavior-focused prevention among rural and socioeconomically disadvantaged groups.

## Data Availability

The raw data supporting the conclusions of this article will be made available by the authors, without undue reservation.

## References

[1] Sakagianni A Koufopoulou C Koufopoulos P Feretzakis G Koumaki V. The impact of COVID-19 on the epidemiology of carbapenem resistance. Antibiotics. (2025) 14:916. doi: 10.3390/antibiotics14090916 41009895 PMC12466860

[2] Baker RE Mahmud AS Miller IF Rajeev M Rasambainarivo F Rice BL . Infectious disease in an era of global change. Nat Rev Microbiol. (2022) 20:193–205. doi: 10.1038/s41579-021-00639-z 34646006 PMC8513385

[3] Zhuang M Zhai L Zhang H Chen Q Xiong R Liu Y . Rural residents' knowledge, attitude, and practice in relation to infection risk during the late stage of an epidemic: a cross-sectional study of COVID-19. Front Public Health. (2024) 12:1450744. doi: 10.3389/fpubh.2024.1450744 39697290 PMC11652518

[4] Sargsyan Z Grigoryan Z Sahakyan S Agopian A Harutyunyan T. Socio-demographic determinants of infectious disease-related health literacy and knowledge in Armenia: results from a nationwide survey. PLoS ONE. (2024) 19:e0307300. doi: 10.1371/journal.pone.0307300 39024390 PMC11257219

[5] Zhao Y Xu S Zhang X Wang L Huang Y Wu S . The effectiveness of improving infectious disease-specific health literacy among residents: wechat-based health education intervention program. JMIR Form Res. (2023) 7:e46841. doi: 10.2196/46841 37556189 PMC10448287

[6] Wang X Zhang X Chen S Shi K Cui W Shi F . Infectious disease-specific health literacy and its influencing factors: Research results based on a cross-sectional design study carried out in Shandong Province's rural areas. Medicine. (2025) 104:e42084. doi: 10.1097/MD.0000000000042084 40193649 PMC11977720

[7] Qin J Gong Y Hu R Lou Y Xuan X Wang P . Associations of infectious disease-specific, electronic, and general health literacy among high school students with prevalent health challenges: a cross-sectional study. Original research. Front Public Health. (2025) 13:1613375. doi: 10.3389/fpubh.2025.1613375 40438059 PMC12117663

[8] Zeng Y Li F Liang W Liu Y Zou Z Baker JS . Knowledge, practice, and information sources regarding infectious diseases among Chinese children and adolescents: a national-level cross-sectional study. BMC Public Health. (2025) 25:412. doi: 10.1186/s12889-025-21516-x 39893404 PMC11787737

[9] Ashok A Acharya PR Mohandas NV Sugathan A Priya A Acharya V . A hospital based cross-sectional analysis of knowledge, attitude and perception of vaccinations among adult patients with chronic respiratory diseases in Karnataka, South India. Clin Epidemiol Glob Health. (2025) 35:102162. doi: 10.1016/j.cegh.2025.102162

[10] Liu B Zhang X Lai Y Sun T Wang C Zhao T . Global vaccine confidence trends among adults above and below age 65. NPJ Vaccines. (2025) 10:160. doi: 10.1038/s41541-025-01217-7 40691165 PMC12280150

[11] Manna A Karsai M Perra N. Social inequalities in vaccine coverage and their effects on epidemic spreading. PLoS Comput Biol. (2025) 21:e1013585. doi: 10.1371/journal.pcbi.1013585 41082566 PMC12533974

[12] Giuffrida A Saia-Owenby C Andriano C Beall D Bailey-Classen A Buchanan P . Social media behavior guidelines for healthcare professionals: an American society of pain and neuroscience NEURON project. J Pain Res. (2024) 17:3587–99. doi: 10.2147/JPR.S488590 39529946 PMC11551221

[13] Denniss E Lindberg R. Social media and the spread of misinformation: infectious and a threat to public health. Health Promot Int. (2025) 40:daaf023. doi: 10.1093/heapro/daaf023 40159949 PMC11955583

[14] Rodrigues F Newell R Rathnaiah Babu G Chatterjee T Sandhu NK Gupta L. The social media infodemic of health-related misinformation and technical solutions. Health Policy Technol. (2024) 13:100846. doi: 10.1016/j.hlpt.2024.100846

[15] Li J Wang Y Zhang Y Liu J Dong Y Xing Y . Strategic co-prevention framework for addressing common health challenges among students in China. Future. (2025) 3:7. doi: 10.3390/future3020007

[16] Owoyemi A Okolie EA Omitiran K Amaechi UA Sodipo BO Ajumobi O . Importance of community-level interventions during the COVID-19 pandemic: lessons from Sub-Saharan Africa. Am J Trop Med Hyg. (2021) 105:879–83. doi: 10.4269/ajtmh.20-1533 34370697 PMC8592170

[17] Su B Talifu Z Feng L. Epidemiological shifts in infectious diseases in China: implications and policy recommendations. China CDC Wkly. (2023) 5:948–51. doi: 10.46234/ccdcw2023.178 38026098 PMC10646164

[18] Zhao ZY Li JJ Ouyang HQ Li WH Huang SK Ohore OE . Enhancing regional disease burden estimates: insights from the comparison of Global Burden of Disease and China's notifiable infectious diseases data with policy implications (2010–2020). Infect Dis Poverty. (2025) 14:81. doi: 10.1186/s40249-025-01351-3 40770780 PMC12326785

[19] Sohail A Cheng AC McGuinness SL Leder K. The epidemiology of notifiable diseases in Australia and the impact of the COVID-19 pandemic, 2012-2022. BMC Glob Public Health. (2024) 2:1. doi: 10.1186/s44263-023-00029-y 39681889 PMC11622879

[20] Sentürk H Borlu A Durmuş H Çetinkaya F. Can health literacy effectively enhance blood donation rates? Indian J Hematol Blood Transfus. (2025) 41:605–12. doi: 10.1007/s12288-024-01872-1 40687465 PMC12267730

[21] Zhao Y Xu Y Yao D Wu Q Chen H Hu X . Changes in infectious disease-specific health literacy in the post-COVID-19 pandemic period: two-round cross-sectional survey study. JMIR Public Health Surveill. (2024) 10:e52666. doi: 10.2196/52666 39213137 PMC11378864

[22] Yang W Liu Y Zhang G Yao Y Wang Y Leng D . Health literacy and associated factors in China: findings from the wa ethnic group. Front Public Health. (2024) 12:1407593. doi: 10.3389/fpubh.2024.1407593 38979042 PMC11228141

[23] Li Z Tian Y Gong Z Qian L. Health literacy and regional heterogeneities in China: a population-based study. Front Public Health. (2021) 9:603325. doi: 10.3389/fpubh.2021.603325 34046382 PMC8144299

[24] Chen S Wang B Wang X Shi K Cui W Liu Y . Study on health education methods based on rural residents' infectious disease-specific health literacy in Shandong, China. Medicine. (2024) 103:e39292. doi: 10.1097/MD.0000000000039292 39121244 PMC11315526

[25] Luo S Xie J Chen J Li H Zhang S. Survey of public knowledge, attitudes, and practices regarding personal protection against COVID-19 in the post-pandemic era. Front Psychol. (2024) 15:1411055. doi: 10.3389/fpsyg.2024.1411055 38915426 PMC11195805

[26] Abu Bakar SF Md Isa Z Ibrahim R Ismail A Daud F Ibrahim R. Knowledge, attitudes, and practices towards COVID-19 prevention among indigenous population in Malaysia: A cross-sectional study. Sci Rep. (2024) 14:21428. doi: 10.1038/s41598-024-72519-4 39271935 PMC11399268

[27] Aron MB Munyaneza F Rosenthal A Dullie L Krumkamp R Ndarama E . Knowledge of local snakes, first-aid and prevention of snakebites among community health workers and community members in rural Malawi: a cross-sectional study. Trop Med Int Health. (2025) 30:84–92. doi: 10.1111/tmi.14071 39686915 PMC11791875

[28] Ramos-Rincón JM Mira-Solves JJ Crespo-Mateos P Oyarzabal M Pérez-Ortiz CI Ramos-Sesma V . Knowledge about chagas disease among family and community medicine residents in a non-endemic region: a cross-sectional study. Am J Trop Med Hyg. (2023) 108:1157–60. doi: 10.4269/ajtmh.22-0081 37160283 PMC10540101

[29] Alwan N Salhab R Hussain F Takshe AA Ghach W. Assessment of hand hygiene awareness and practice levels amid the COVID-19 period: a comparative study of the public in Jordan and the United Arab Emirates. BMC Public Health. (2025) 25:3397. doi: 10.1186/s12889-025-24554-7 41063049 PMC12505845

[30] Hong Q Xu Y. Evaluating hand hygiene knowledge, attitudes, and practices among healthcare workers in post-pandemic H1N1 influenza control: a cross-sectional study from China. Front Public Health. (2024) 12:1432445. doi: 10.3389/fpubh.2024.1432445 39399703 PMC11466785

