# Supplementary material for: Current status of knowledge on the prevention of common infectious diseases among urban and rural populations: a cross-sectional study
Source: Front Public Health. 2026 May 11;14:1771435. doi: 10.3389/fpubh.2026.1771435 (PMC13199263; doi:10.3389/fpubh.2026.1771435)
Supplement: Supplementary file 1 [file Supplementary_File_1.docx]

Supplementary Table 1. Structure and content of the questionnaire assessing knowledge, attitudes, behaviors, and information sources related to the prevention of common infectious diseases among urban and rural residents.

| Section | Item code | Question | Response format | Example categories / coding* |
| --- | --- | --- | --- | --- |
| Sociodemographic information | A1 | What is your age (in completed years)? | Open numeric | Years (e.g., 18–95) |
| Sociodemographic information | A2 | What is your sex? | Single choice | 1 = Male; 2 = Female; 3 = Other |
| Sociodemographic information | A3 | What is your highest level of education completed? | Single choice | 1 = Primary school or below; 2 = Junior high; 3 = Senior high/vocational; 4 = College or above |
| Sociodemographic information | A4 | What is your current occupation? | Single choice (or open, then recoded) | 1 = Farmer; 2 = Worker; 3 = Office staff; 4 = Self-employed; 5 = Student; 6 = Unemployed/retired; 7 = Other |
| Sociodemographic information | A5 | Where do you currently live? | Single choice | 1 = Urban; 2 = Rural |
| Sociodemographic information | A6 | What is your current marital status? | Single choice | 1 = Single; 2 = Married/cohabiting; 3 = Divorced; 4 = Widowed |
| Sociodemographic information | A7 | What is your approximate monthly household income? | Single choice | 1 = Below local average; 2 = Around local average; 3 = Above local average; 4 = Not sure / refuse to answer |
| General knowledge of infectious diseases | B1 | Infectious diseases are illnesses caused by pathogenic microorganisms (such as bacteria or viruses). | Single choice (knowledge) | 1 = True; 2 = False; 3 = Do not know |
| General knowledge of infectious diseases | B2 | Common infectious diseases can spread from person to person. | Single choice | 1 = True; 2 = False; 3 = Do not know |
| General knowledge of infectious diseases | B3 | Some infectious diseases may have no obvious symptoms in the early stage. | Single choice | 1 = True; 2 = False; 3 = Do not know |
| General knowledge of infectious diseases | B4 | Once infected, all infectious diseases are impossible to prevent from spreading to others. | Single choice | 1 = True; 2 = False; 3 = Do not know |
| General knowledge of infectious diseases | B5 | Early diagnosis and treatment can reduce the complications of many infectious diseases. | Single choice | 1 = True; 2 = False; 3 = Do not know |
| General knowledge of infectious diseases | B6 | Vaccination is one of the important measures to prevent certain infectious diseases. | Single choice | 1 = True; 2 = False; 3 = Do not know |
| Routes of transmission | C1 | Some infectious diseases can be transmitted through close contact with an infected person. | Single choice | 1 = True; 2 = False; 3 = Do not know |
| Routes of transmission | C2 | Some infectious diseases can be transmitted through contaminated food or drinking water. | Single choice | 1 = True; 2 = False; 3 = Do not know |
| Routes of transmission | C3 | Coughing or sneezing can spread infectious agents through respiratory droplets. | Single choice | 1 = True; 2 = False; 3 = Do not know |
| Routes of transmission | C4 | Mosquitoes, ticks, or other vectors can transmit certain infectious diseases to humans. | Single choice | 1 = True; 2 = False; 3 = Do not know |
| Routes of transmission | C5 | Sharing needles or syringes with others may increase the risk of infection with blood-borne diseases. | Single choice | 1 = True; 2 = False; 3 = Do not know |
| Knowledge of preventive measures | D1 | Frequent handwashing with soap and running water can help prevent many infectious diseases. | Single choice | 1 = True; 2 = False; 3 = Do not know |
| Knowledge of preventive measures | D2 | Wearing a mask in crowded or poorly ventilated places can reduce the risk of respiratory infections. | Single choice | 1 = True; 2 = False; 3 = Do not know |
| Knowledge of preventive measures | D3 | Properly cooking food and drinking safe water can reduce the risk of intestinal infectious diseases. | Single choice | 1 = True; 2 = False; 3 = Do not know |
| Knowledge of preventive measures | D4 | Vaccination according to national immunization schedules helps prevent certain infectious diseases. | Single choice | 1 = True; 2 = False; 3 = Do not know |
| Knowledge of preventive measures | D5 | Maintaining good indoor ventilation and environmental sanitation helps reduce the spread of infectious diseases. | Single choice | 1 = True; 2 = False; 3 = Do not know |
| Knowledge of preventive measures | D6 | Avoiding close contact with people who have fever or respiratory symptoms can help prevent infection. | Single choice | 1 = True; 2 = False; 3 = Do not know |
| Risk-related behaviors and attitudes | E1 | Have you ever received any vaccines recommended for adults (for example, influenza or other vaccines)? | Single choice (behavior) | 0 = No; 1 = Yes; 9 = Not sure / do not remember |
| Risk-related behaviors and attitudes | E2 | When you have fever, cough, or diarrhea that persists, how likely are you to seek care at a medical institution? | 5-point Likert scale (attitude) | 1 = Very unlikely; 2 = Unlikely; 3 = Uncertain; 4 = Likely; 5 = Very likely |
| Risk-related behaviors and attitudes | E3 | In daily life, how often do you wash your hands with soap before eating or after using the toilet? | 5-point Likert scale (behavior) | 1 = Never; 2 = Rarely; 3 = Sometimes; 4 = Often; 5 = Always |
| Risk-related behaviors and attitudes | E4 | In crowded indoor places during epidemic seasons, how often do you wear a mask? | 5-point Likert scale (behavior) | 1 = Never; 2 = Rarely; 3 = Sometimes; 4 = Often; 5 = Always |
| Risk-related behaviors and attitudes | E5 | I am willing to follow public health recommendations to prevent the spread of infectious diseases. | 5-point Likert scale (attitude) | 1 = Strongly disagree; 2 = Disagree; 3 = Neutral; 4 = Agree; 5 = Strongly agree |
| Risk-related behaviors and attitudes | E6 | I believe that my own behaviors (such as handwashing and mask use) can effectively reduce the risk of infection. | 5-point Likert scale (attitude) | 1 = Strongly disagree; 2 = Disagree; 3 = Neutral; 4 = Agree; 5 = Strongly agree |
| Sources of information | F1 | In the past year, from which channels have you obtained information about infectious disease prevention? ** | Multiple choice (tick all that apply) | 1 = Television/radio; 2 = Newspapers/magazines; 3 = Internet websites; 4 = Social media; 5 = Healthcare professionals; 6 = Community health education; 7 = Family/friends; 8 = Other |
| Sources of information | F2 | Which channel do you consider the most reliable source of information on infectious disease prevention? | Single choice | Same options as F1, choose one main source |
| Sources of information | F3 | How satisfied are you with the infectious disease prevention information you currently receive? | 5-point Likert scale | 1 = Very dissatisfied; 2 = Dissatisfied; 3 = Neutral; 4 = Satisfied; 5 = Very satisfied |

* Coding indicates the numeric values assigned for data entry and statistical analysis. For knowledge items (Sections B–D), responses were coded as 1 = correct answer, 0 = incorrect or “Do not know.” For other sections, coding values reflect categorical response options as listed.

** For multiple-choice items, participants were allowed to select more than one option. Each option was coded as a separate binary variable (0 = not selected; 1 = selected) for analysis.

Correct answers for knowledge items (Sections B–D):

B1 = True

B2 = True

B3 = True

B4 = False

B5 = True

B6 = True

C1 = True

C2 = True

C3 = True

C4 = True

C5 = True

D1 = True

D2 = True

D3 = True

D4 = True

D5 = True

D6 = True

Each correct response was awarded one point. The total knowledge score ranged from 0 to 17.

Supplementary Table S2. Ten-fold cross-validation performance

| Fold | AUC |
| --- | --- |
| 1 | 0.72 |
| 2 | 0.74 |
| 3 | 0.73 |
| 4 | 0.71 |
| 5 | 0.75 |
| 6 | 0.72 |
| 7 | 0.74 |
| 8 | 0.73 |
| 9 | 0.72 |
| 10 | 0.74 |
| Mean ± SD | 0.73 ± 0.01 |
